# A condition-specific codon optimization approach for improved heterologous gene expression in *Saccharomyces cerevisiae*

**DOI:** 10.1186/1752-0509-8-33

**Published:** 2014-03-17

**Authors:** Amanda M Lanza, Kathleen A Curran, Lindsey G Rey, Hal S Alper

**Affiliations:** 1 Department of Chemical Engineering, The University of Texas at Austin, 200 E Dean Keeton St. Stop C0400, Austin, TX 78712, USA; 2 Institute for Cellular and Molecular Biology, The University of Texas at Austin, 2500 Speedway Avenue, Austin, TX 78712, USA; 3 Current Address: Bristol-Myers Squibb, Biologics Development, 35 South Street, Hopkinton, MA 01748, USA

**Keywords:** Codon optimization, Codon bias, *Saccharomyces cerevisiae*, Heterologous expression, Synthetic biology

## Abstract

**Background:**

Heterologous gene expression is an important tool for synthetic biology that enables metabolic engineering and the production of non-natural biologics in a variety of host organisms. The translational efficiency of heterologous genes can often be improved by optimizing synonymous codon usage to better match the host organism. However, traditional approaches for optimization neglect to take into account many factors known to influence synonymous codon distributions.

**Results:**

Here we define an alternative approach for codon optimization that utilizes systems level information and codon context for the condition under which heterologous genes are being expressed. Furthermore, we utilize a probabilistic algorithm to generate multiple variants of a given gene. We demonstrate improved translational efficiency using this condition-specific codon optimization approach with two heterologous genes, the fluorescent protein-encoding *eGFP* and the catechol 1,2-dioxygenase gene *CatA*, expressed in *S. cerevisiae*. For the latter case, optimization for stationary phase production resulted in nearly 2.9-fold improvements over commercial gene optimization algorithms.

**Conclusions:**

Codon optimization is now often a standard tool for protein expression, and while a variety of tools and approaches have been developed, they do not guarantee improved performance for all hosts of applications. Here, we suggest an alternative method for condition-specific codon optimization and demonstrate its utility in *Saccharomyces cerevisiae* as a proof of concept. However, this technique should be applicable to any organism for which gene expression data can be generated and is thus of potential interest for a variety of applications in metabolic and cellular engineering.

## Background

Codon optimization is commonly used to improve heterologous gene expression, especially in the context of synthetic biology and metabolic and cellular engineering [1,2]. While most commonly employed in prokaryotic systems [3-5], codon optimization has also been described in eukaryotic systems such as yeast [1,6-8]. The basic premise behind this approach is that the distribution of the 64 unique DNA codons is non-random. Specifically, the occurrence of synonymous codons (i.e. different codons all encoding for the same amino acid) within any genome is not uniform, resulting in both rare and abundant codons. The distribution of preferred codons varies across all organisms [1,9,10] giving rise to a host-specific codon usage bias (CUB) [11]. Codon usage, especially the high prevalence of rare codons, is known to influence translational efficiency [12]. As a result, the most common strategy for codon optimization is to replace rare codons with more frequently occurring ones, thereby matching the CUB of the host organism.

Typically, the CUB for a given organism is determined using the Codon Usage Tabulated from GenBank (CUTG) [13]. This process calculates the frequency of codon usage across all annotated protein coding genes and is the primary dataset used for codon optimization. Several alternative codon usage tables exist including the codon adaptation index (CAI) [14,15], codon bias index (CBI) [16] and effective number of codons (N_c_) [17]. As an outgrowth of these approaches, several online optimization programs have been developed and are freely available [18-20]. Despite the promise of these approaches, much of this research has focused on studying and describing endogenous gene expression. Moreover, traditional codon optimization does not always lead to improved expression compared to a wild-type, unmodified sequence [21-23]. In fact, in a survey of 44 synthetic genes manufactured by Blue Heron Biotechnology (http://www.blueheronbio.com/assets/documents/BlueHeronBioExpressionSurvey.pdf), 32% of the “optimized” synthetic genes expressed at lower levels than the wild-type. Thus, alternative strategies are required to further improve codon optimization for heterologous genes, especially in eukaryotic hosts and for biotechnological applications.

We posit that the limitations to traditional codon optimization stem from the fact that traditional methods for CUB calculations utilize all potential coding regions of the genome. It is well documented that tRNA abundance is influenced by changes in environmental factors including growth condition and cell-cycle [24-27]. Moreover, much of an organism’s protein coding genes are lowly expressed and thus minimal evolutionary pressure has been present to drive efficient natural evolution and optimization [11,28]. As a result, it is expected that the effective CUB for a given growth condition may differ from the whole-genome based CUB. Thus, a traditional codon optimization approach neglects cell conditions and considers all of a genome’s protein coding information as equal. As a result, these approaches fail to capture important nuances of the effective CUB that may be essential for guaranteeing expression of a heterologous gene. In addition, traditional approaches neglect evidence that adjacent codons co-evolve [29-31]. Finally, it is not always wise to consistently utilize abundant codons, especially in biotechnological applications where multiple genes are optimized and the cognate tRNA may eventually become limiting [27].

Here we demonstrate that a CUB generated using only genes expressed under a given condition can enable improved codon optimization in *Saccharomyces cerevisiae* compared to a CUB generated using the CUTG. We refer to this alternative approach as ‘condition-specific codon optimization.’ Furthermore, we utilize a probabilistic method that incorporates codon context into optimized gene design and thus results in multiple variants to be tested rather than a singular design. We demonstrate the utility of this technique in *S. cerevisiae* through the condition-specific codon optimization of two heterologous genes: a green fluorescent protein variant originally optimized for expression in *Escherichia coli*, and the catechol 1,2-dioxygenase enzyme from *Acinetobacter baylyi*. To do so, we created a CUB for two specific conditions of interest: constitutive high expression and high expression in stationary phase growth. The resulting optimized genes yielded more protein activity under the conditions for which they were optimized. Furthermore, our best catechol 1,2-dioxygenase gene variant resulted in 2.9-fold higher activity than a commercially optimized gene variant. This technique should be applicable to any organism for which gene expression data can be generated and is thus of potential interest for a variety of applications in metabolic and cellular engineering.

## Results and discussion

### Developing a condition-specific codon usage bias matrix

**Figure 1:**
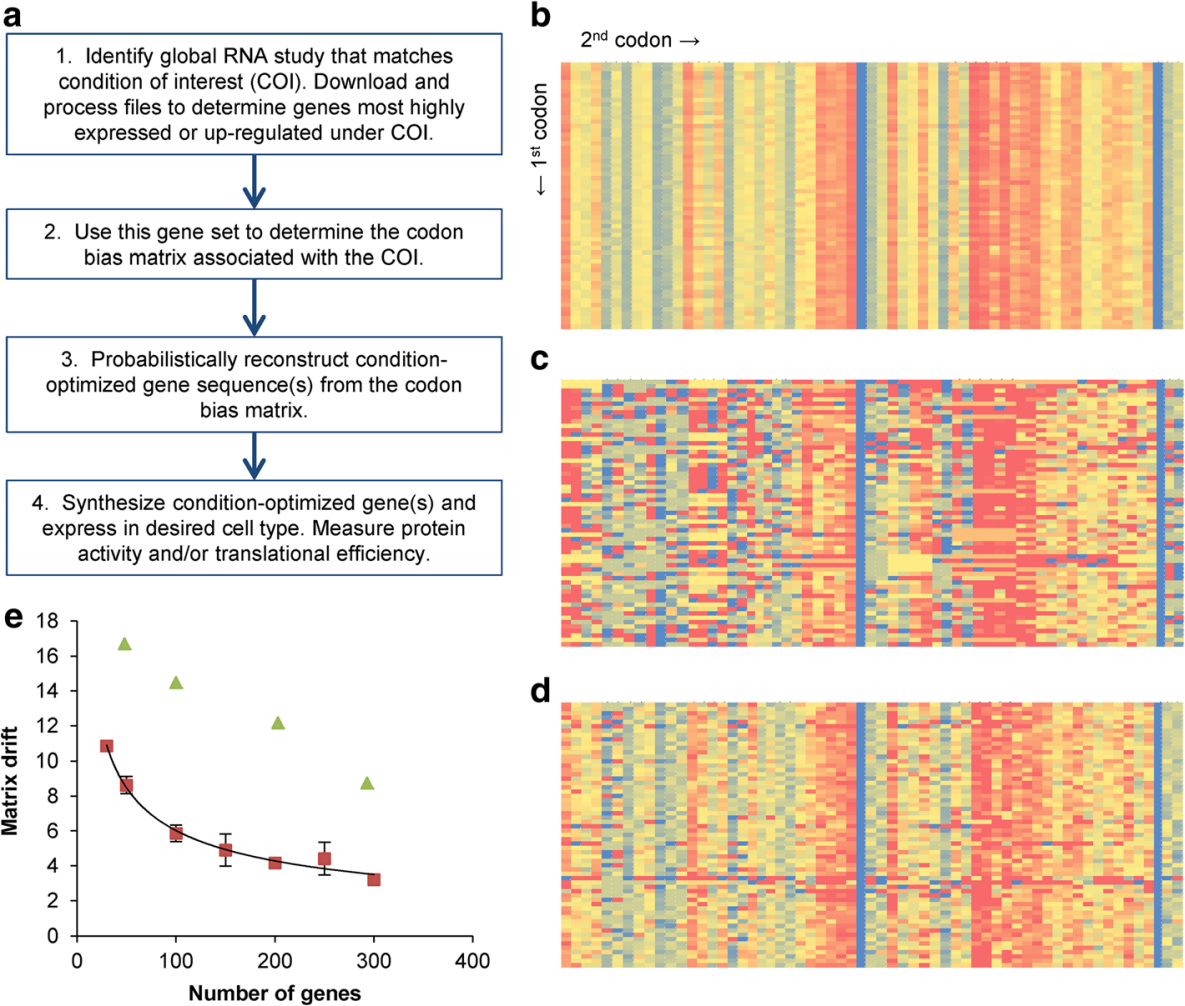
**Condition-specific codon optimization utilizing systems level information and codon context. a**. A generic workflow to enable a condition-specific codon optimization algorithm in any organism from gene expression data. **b**. The control codon matrix is compiled from all 6,666 protein-coding genes in *S. cerevisiae* and serves as a point of comparison for condition-specific matrices. The first amino acid is indicated by the first column, and the second amino acid by the first row. The color indicates probability between 0 (red) and 1 (blue). **c**. The high expression codon optimization matrix is compiled from the 100 most highly expressed protein-coding genes in *S. cerevisiae*[35]*.***d**. The stationary phase codon optimization matrix is compiled from the 50 most highly expressed protein-coding genes in *S. cerevisiae* grown for 3 days, compared to an exponential population [38]. **e**. The matrix drift from the control matrix (as indicated by Frobenius matrix norm) versus number of genes used to generate the codon usage matrices was plotted for codon usage matrices generated from a random sampling of genes (red squares) and the most highly expressed genes [35] (green triangles). The random data sets were fit with a power regression model. Standard deviations from five independent samples were used to generate error bars.

To establish condition-specific codon optimization, we developed a simple workflow for optimizing heterologous genes (outlined in Figure 1a).

First, the condition under which the heterologous gene of interest will be expressed must be identified and genome-scale expression data for the host should be obtained under this condition. While we have selected DNA microarray data for this study, it is conceivable to use additional sources such as RNA-seq or proteomics. For commonly studied organisms, the desired information is publically available in databases such as Gene Expression Omnibus (GEO), the Center for Information Biology Gene Expression database (CIBEX) and Array Express.

Second, using the appropriate dataset, genes that are differentially up-regulated or highly expressed under the desired condition are identified. From this set of genes and their corresponding DNA sequences, codon frequency and probability can be determined for codon pair usage (codon context). The python script used to generate the condition-specific tables and matrices is entitled ‘CodonUsageAnalysis’ (see Additional file 1). The output of this analysis is a 61 by 61 matrix termed the ‘codon bias matrix’ (the three stop codons were excluded from this matrix). We chose to focus on codon context rather than individual codons as previous studies suggest that codon context may be more important for gene optimization [29-32] and that this context directly correlates with translation elongation rate [33]. In particular, steric hindrance of charged tRNAs by adjacent codons can be avoided by considering the impact of adjacent codon pairing [30].

Third, the condition-specific matrix can be used to reconstruct a series of codon optimized genes that would follow the codon bias rules of the condition of interest. By considering each adjacent codon pairing in the chain, we probabilistically reconstruct the DNA sequence from the protein sequence utilizing the codon context probabilities stored in the condition-specific matrix (performed via a python script entitled ‘GeneDesign’ included as Additional file 2). As an example, if the first two amino acids of the protein sequence are methionine followed by cysteine, there are two possible corresponding DNA sequences: ATGTGT and ATGTGC. The condition-specific matrix stores the probability in which each pairing occurred in the set of up-regulated genes for a given condition. GeneDesign assigns a DNA sequence based on the corresponding probability. For example, if 60% of Met-Cys pairs are ATGTGT and only 40% are ATGTGC, GeneDesign will probabilistically select ATGTGT 60% of the time and ATGTGC 40% of the time for each occasion that a Met-Cys pair is present in the peptide sequence. As a result of the probabilistic design, several variants of codon optimized genes are generated. Moreover, this process also ensures a balance in codon usage rather than the exclusive use of specific codons which can result in bottlenecks with the formation of charged tRNA-amino acid complexes and reduced translational efficiency [27].

Finally, after GeneDesign has been used to generate one or more condition-specific codon optimized sequences, the corresponding DNA can be synthesized and introduced into the cellular host of interest. Functional assays can be used to determine the highest performing variant.

### Generation of condition-specific codon bias matrices for *S. cerevisiae*

In order to validate our hypothesis and this approach, we selected two heterologous genes of interest, applied the approach as outlined above, expressed the resulting optimized genes in *S. cerevisiae*, and measured resulting protein activity. For comparison, we generated a control table and codon context matrix (hereafter referred to as the control matrix), which were assembled using the protein coding sequences of 6,666 *S. cerevisiae* genes. The control table is identical to the CUTG from GenBank for *S. cerevisiae,* which is used commercially. A colored representation of the control matrix is shown in Figure 1b (see Additional file 3 for full data set) with the y-axis representing the first codon and the x-axis representing the second codon in a pair. Each square represents the probability of a codon pair occurring given that the first codon is specified, with the color blue representing a probability of one and red representing a probability of zero. The two solid blue columns correspond to a second codon for methionine (ATG) or tryptophan (TGG), both of which have no synonymous codons and therefore have a probability of 1. Amongst the synonymous codons however, there is variation between the columns, indicating an overall genomic preference for particular synonymous codons. There is very little variation between rows, indicating that the choice in second codon is not influenced much by the choice in first codon. Since this control matrix incorporates codon context for nearly all protein coding genes in *S. cerevisiae*, the probability values are an average of codon usage across the entire genome. As a result, the columns become indicative of the frequency of each codon, with the rarest indicated in red.

In contrast, the matrices made for the subset of highest expressed genes and genes expressed in stationary phase (Figures 1c-1d, respectively) have significant variation between rows (see Additional file 3 for full data set). In order to compare the difference between the control matrix and condition-specific matrices we quantified the drift using the Frobenius matrix norm of the difference between the matrices. This metric is well established as a quantitative tool to determine drift between matrices of the same size [34]. A smaller Frobenius matrix norm indicates higher similarity between matrices, such that identical matrices have a norm of zero. We first evaluated the drift between the control matrix and sets of randomly selected genes as a control. Random sets of 30, 50, 100, 150, 200, 250 and 300 genes were selected in five independent events and the average matrix norm was calculated (Figure 1e). As the number of randomly considered genes increases, the Frobenius matrix norm or drift relative to the control condition decreases in a power regression fashion (of the form y = Ax^b^). This behavior is expected, as the inclusion of more genes in a codon usage matrix will result in an averaging effect that begins to resemble the control matrix composed of all gene sequences. Next, we calculated the drift between the control matrix and the highest expressed genes in *S. cerevisiae*[35]. The higher drift demonstrates that these genes exhibit a vastly different codon usage than the control matrix. The difference in codon usage between highly expressed genes and the rest of the genome has been previously noted [16], suggesting evolutionary pressure on codon usage. This further supports the use of only a subset of genes for determining codon usage bias.

### Condition-specific optimization of *eGFP* for high expression outperforms wild-type and control variants

**Figure 2:**
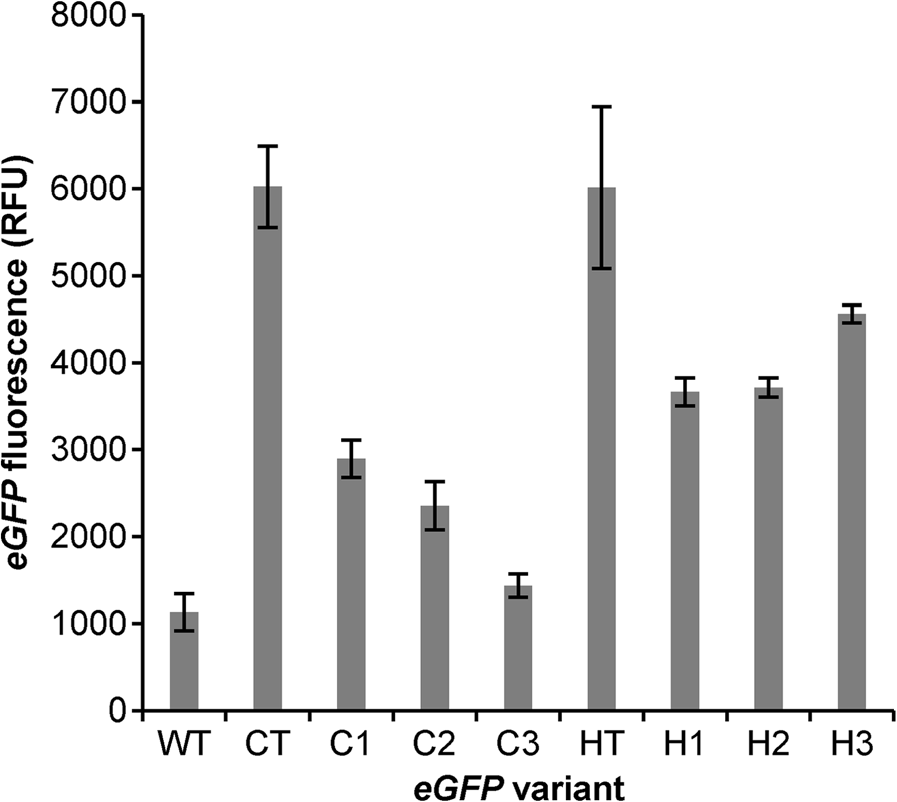
**Optimization for a high expression condition results in *eGFP *expression exceeding the wild-type.** In addition to wild-type *eGFP*, eight variants were generated. The high expression variants were made from a codon usage table (HT) and matrix (H1-H3) constructed using the 100 most highly expressed genes in yeast grown in rich media. Control variants were constructed from the standard usage table (CT) and control matrix (C1-C3). *eGFP* protein expression was measured using flow cytometry for yeast grown in YPD. Biological triplicates were used to calculate standard deviations, indicated by error bars and p-values were calculated using a t-test to determine statistical significance (described in text of paper).

Initially, we sought to investigate the importance of condition-specific codon optimization by re-coding an *E. coli* fluorescence protein for high, constitutive expression in yeast. To do so, we established a CUB based on the 100 most highly expressed genes during growth in YPD media [35]. The associated condition-specific table and matrix were assembled as described above (Figure 1c, see Additional files 3 and 4 for full matrices and tables, respectively). An *E. coli* optimized green fluorescent protein (*eGFP*) was selected as a reporter protein as it is poorly translated in yeast. Eight sequence variants of *eGFP* were generated and compared to the wild-type sequence. One variant was optimized using the control table, one variant using the high expression table, three variants using probabilistic design based on the control matrix and three variants using probabilistic design based on the high expression matrix. The sequences for each of these variants can be found in Additional file 4. Each variant, including wild-type *eGFP*, was inserted into the p41K-GPD yeast expression vector and transformed into *S. cerevisiae* BY4741 [36] and fluorescence was screened in mid-log phase of YPD growth using biological triplicates. We observed that, on average, the *eGFP* variants generated using the high expression matrix are statistically better expressed than those variants generated using the control matrix (p-value = 6.1e-6) and better than the wild-type *eGFP* (p-value = 1.4e-6) (Figure 2). While all three of the high expression matrix-generated variants outperform the wild-type *eGFP*, only two of the three control matrix-generated variants outperform wild-type *eGFP*. These results demonstrate that optimizing codon usage specifically for high expression was effective and that the probabilistic design is suitable for generating functional variants.

We also tested the *eGFP* variants generated using the control and high expression table rather than a probabilistic matrix-based design. While there was no statistical difference between the two conditions, expression of these variants were higher than any of the matrix-generated conditions. This result is not surprising given the short length of the gene (251 amino acids). However, a table-optimized approach lacks codon diversity which may ultimately become a bottleneck and decrease enzyme fitness for large metabolic engineering endeavors with multiple genes [23,27,37]. The fact that two table-optimized genes were similar is not unexpected either, as there was a difference between the most abundant codon for only six of the 20 amino acids in the two tables, and thus the genes codon optimized by both tables were highly similar on a sequence basis. Nevertheless, these results illustrate that the probabilistic method used to choose codons from the condition specific matrix gives better results than that from the control matrix. As such, this is the first proof-of-concept demonstrating that using condition-specific data can improve codon optimization gene design over the traditional CUB methods.

### High expression and stationary phase optimization of *CatA*

**Figure 3:**
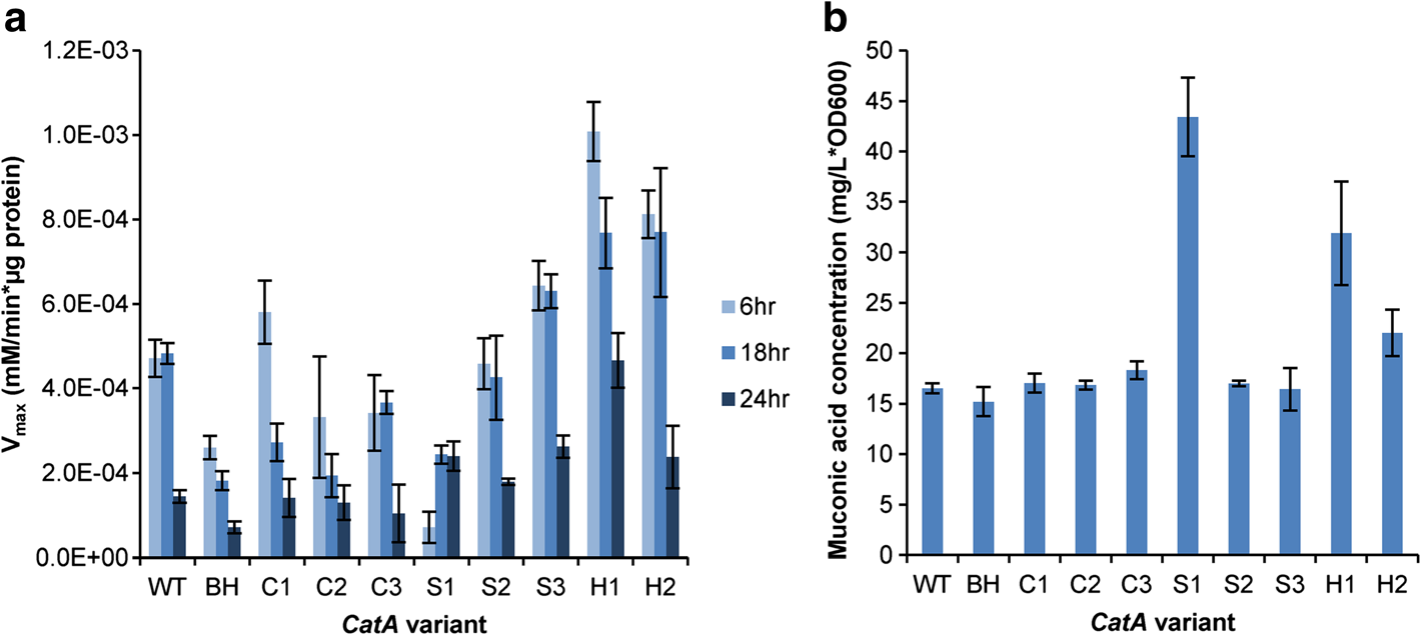
**Optimization for stationary phase results in *CatA *variants that are improved at late growth.** Ten *CatA* variants were generated, including wild-type and a version optimized by Blue Heron Biotechnology. The three stationary phase variants (S1-S3) were made from a codon usage matrix constructed using the 50 most highly expressed genes after three days of growth (see Supplementary Matrices). The two high-expression variants (H1-H2) were made from a codon usage matrix constructed using the 100 most highly expressed genes in yeast grown in rich media. The three control variants (C1-C3) were constructed from the control matrix). Two assays were conducted to measure activity. **a**. Cells expressing the *CatA* variants were grown for 6, 18 or 24 hours prior to bulk protein extraction. The V_max_ (mM/min*μg protein) for conversion of catechol to muconic acid was determined for the bulk protein. Biological triplicates and technical triplicates were measured to determine standard deviations. **b**. Cells expressing the *CatA* variants were grown for 18 hours in 30 mL before spiking the media with 1 g/L of catechol. After 24 additional hours of growth, 1 mL of supernatant was extracted and analyzed using HPLC, as previously described [22], to determine total muconic acid production. Normalized muconic acid levels (mg/L*OD_600_) are reported and standard deviation was determined using biological triplicates and p-values were calculated using a t-test to determine statistical significance (described in text of paper).

Next, we sought to codon optimize a catechol 1,2-dioxygenase gene (*CatA*) from *Acinetobacter baylyi* in *S. cerevisiae* to enable the production of muconic acid, a useful polymer precursor, in the stationary phase. This condition was chosen as it is often desirable to delay gene expression until stationary phase to increase product output and separate growth and production phases. To optimize under these conditions, a codon usage matrix was calculated using the 50 genes most differentially upregulated when comparing three day cultures to exponential growth [38] (Figure 1d, see Additional file 3 for full matrices). This data was downloaded from a previous study available under the GEO reference E-TABM-496. We designed three *CatA* variants using a probabilistic design based on the stationary phase matrix (named stationary #1 through #3), three similarly designed using the control matrix (named control #1 through #3), and two using the high expression matrix used above for *e*GFP (named high expression #1 through #2). Finally, we included wild-type *A. baylyi CatA* and a variant that was codon-optimized for expression in *S. cerevisiae* by Blue Heron Biotechnology using traditional codon optimization methods (referred to as the Blue Heron variant). These sequences are included in Additional file 4. Expression of these ten variants was determined using a previously described protein activity assay [22] during various stages of growth (6, 18 and 24 hours post inoculum). We calculated the catalytic rate, V_max_ (mM/min*μg protein), for each variant (Figure 3a).

In exponential phase (6 hours of growth), the V_max_ for wild-type *CatA* is significantly higher than the Blue Heron variant (p-value = 0.002). This illustrates again that traditional codon optimization approaches can often result in poor performance. The most highly expressed variant after 6 hours of growth is high expression #1 whereas the lowest expressed variant is stationary #1.

Toward late exponential phase and early stationary phase (18 hours), the *CatA* expression pattern shifts for each variant. Compared to the 6 hour time-point, the cellular catalytic level (V_max_) at 18 hours decreases for many of the constructs including the control variants #1 and #2, high expression #1, and the Blue Heron variant. By comparison, V_max_ for stationary #2 and #3 is unchanged between 6 and 18 hours, and for stationary #1, V_max_ actually increases. At 18 hours, the average V_max_ for the stationary variants is significantly higher than the average for the control variants (p-value = 0.013) and the Blue Heron variant (p-value = 0.026). While the stationary phase variants either maintain or increase their V_max_, the activity of the control variants decreases significantly. Moreover, at this timepoint, the control variants perform worse than the *A. baylyi* wild-type gene.

The disparity between the average V_max_ for the stationary and control variants is even more significant (p-value = 4.7e-4) at 24 hours. Furthermore, outside of high expression variant #1, the stationary variants #1 and #3 demonstrate the highest V_max_ values. The profile for stationary #1 is particularly interesting as it was among the worst during exponential phase and among the best at stationary phase. These results provide further demonstration that codon optimization based on condition-specific CUBs can outperform traditional approaches. By doing so, we were able to design three *CatA* variants which outperformed both a commercially-optimized sequence (Blue Heron variant) and three control *CatA* variants in stationary phase.

We have previously demonstrated that catalytic capacity is just as important as catalytic rate for the *CatA* enzyme [22]. To measure this, cultures with each variant were grown for 18 hours and then spiked with 1 mg/ml catechol. Resulting muconic acid (normalized to cell count) was measured via HPLC for each of the variants (Figure 3b). In this assay, the stationary #1 variant outperformed all other variants, including the high expression #1 variant (p-value = 0.036). Overall, this variant had a catalytic capacity that was 2.6-fold higher than the wild-type version and nearly 2.9-fold higher than the Blue Heron optimized version in the stationary phase—the condition used to optimize this gene. These results highlight the importance and potential of condition specific codon optimization, and demonstrate for the first time that codon optimization can be used to control translation for specific environmental conditions.

### Analysis of transcription factor-regulated genes suggests codon usage is linked to gene regulation

**Figure 4:**
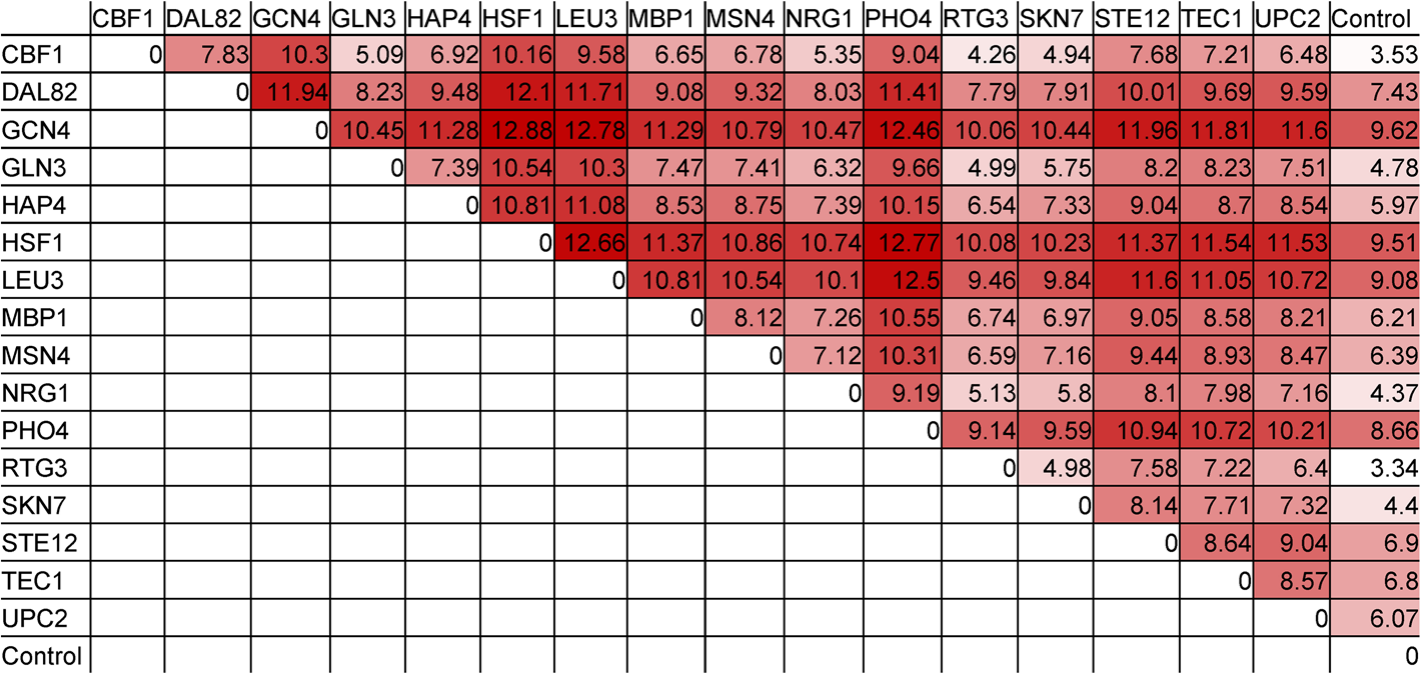
**Drift of transcription-factor codon matrices reveals diverse codon usage relative to the control matrix.** The genetic interaction targets for sixteen *S. cerevisiae* transcription factors were identified using the *Saccharomyces* Genome Database (http://yeastgenome.org). Using those corresponding gene target sequences, codon usage matrices were constructed for each transcription factor. Frobenius matrix norms were calculated for all matrix pairs, including the control matrix (Figure 1b) using MATLAB. The Frobenius norms represent drift between matrices and darker colored cells represent higher drift. A value of zero means the matrices are identical.

Based on the utility of condition-specific CUBs, we were interested in exploring the potential link between transcriptional and translational regulation. Utilizing a systems biology perspective, we concentrated on key global transcription factors in yeast. Specifically, we sought to calculate the CUB matrices for genes regulated by sixteen global transcription factors for *S. cerevisiae*: Cbf1p, Dal82p, Gcn4p, Gln3p, Hap4p, Hsf1p, Leu3p, Mbp1p, Msn4p, Nrg1p, Pho4p, Rtg3p, Skn7p, Ste12p, Tec1p, and Upc2p. Codon usage matrices for the gene targets regulated by each of these global regulators were compared with the control matrix derived from all 6,666 protein coding genes (see Additional file 3 for all matrices). The Frobenius matrix norm was calculated for all pairwise combinations of the 17 matrices (control and 16 transcription factors) and this data set was used to generate a colored table, with darker colored cells representing a higher Frobenius matrix norm, and thus more distinct matrices (Figure 4).

The matrices for Rtg3p targets and Cbf1p targets are both most similar to the control matrix whereas Gcn4p and Hsf1p targets are most dissimilar in their codon usage compared with the control dataset. In general, Figure 4 clearly shows that the genetic targets of transcription factors have very disparate codon usage compared to the aggregate of coding regions. Furthermore, each transcription factor matrix is more similar to the control condition than to another transcription factor matrix. This result further supports the averaging effect seen by using all protein coding genes to create CUB tables, as opposed to a subset. The fact that distinct patterns in codon usage appear for each transcription factor suggests that genetic regulation has co-evolved with codon usage. This finding supports the central hypothesis of this work – that transcriptional profiles (in the form of gene expression data) can be used to predict optimization schemes for translational regulation (in the form of codon optimization).

## Conclusions

Here, we demonstrate the utility of a condition-specific codon optimization method that utilizes both systems-level information and codon context to generate a set of potential variants using a probabilistic algorithm. While we demonstrated the effectiveness of this algorithm using yeast and microarray data, the approach should be generic and easily adaptable to other hosts and other high-throughput datasets. While the consideration of codon pair bias and probabilistic design to create variants has been demonstrated previously [30,32], this is the first example to demonstrate that gene expression data can be used to generate high-expressing variants for a specific condition. In the case of the *CatA* gene, the S1 variant was high-expressing in stationary phase and low-expressing in exponential phase, resulting in the highest productivity of muconic acid of all of the variants. This is of considerable interest as it offers a previously unidentified method of control at the translational regulation level for heterologous gene expression.

It should also be noted that this is the first work to study a specific codon optimization strategy in *S. cerevisiae* in depth. Previous codon optimization strategies have been primarily validated using prokaryotes [3,11,30,37], endogenous expression data [14,15,17], or limited heterologous gene variants [7,39]. In contrast, we expressed two heterologous genes in *S. cerevisiae*, *eGFP* and *CatA*, which were each optimized for different growth conditions. In each case, we observe improved protein expression using the condition-specific optimization technique outlined here. Specifically, *eGFP* expression was successfully optimized for high expression in rich media, and the *CatA* enzyme was optimized for high expression in stationary phase growth, with the highest expression variant resulting in 2.9-fold higher product yield over a commercially optimized variant. Finally, we demonstrated that transcriptional regulation is indeed linked to translational efficiency through the significantly different codon utilization patterns of distinctly regulated genes.

Codon optimization is an important synthetic biology tool that enables recombinant DNA expression. The algorithm we define here is simple to execute and host agnostic. As our ability and desire to produce chemicals in a renewable and environmentally-friendly capacity increases [40,41], the necessity for optimized heterologous expression will likewise increase. Furthermore, many of these processes will be carried out under diverse, non-standard environmental conditions, including changes in temperature, pH, mineral concentration, carbon source, oxygen level, and cell growth phase. The condition-specific approach to codon optimization described here will be a key tool to identify gene variants with the ideal codon usage for any particular condition.

## Methods

### Microarray data analysis

Codon usage profiles were assembled using publicly available microarray data, downloaded from the Gene Expression Omnibus (http://www.ncbi.nlm.nih.gov/geo/). Data pre-processing and normalization was performed using the Robust Multichip Average algorithm [42-44], and Bioconductor's Affy package in R version 2.15.1. Differentially expressed genes were identified using the Linear Models for Microarray Data (LIMMA) package. Probe sets were matched with *S. cerevisiae* genes using information included in Affymetrix's Expression Console Software. Genes with an adjusted p-value less than 0.05 and a log-fold change greater than one or less than negative one were considered differentially expressed. A subset of differentially expressed genes (typically 50) was used to generate a condition-specific codon usage table and matrix, as described in the Results and discussion.

### Plasmid construction

Yeast expression vectors were propagated in *Escherichia coli*. All experiments were conducted using *S. cerevisiae* strain BY4741. The sequences of all genes used in this study are available in Additional file 4. The wild-type and Blue Heron Biotechnology-optimized *CatA* variants were taken from a previous study [22]. All other *CatA* variants were assembled using IDT’s gBlocks. The ten *CatA* sequences were assembled in the p413-TEF vector [45]. The wild-type *eGFP* gene was amplified from the pZE-eGFP [46] plasmid using primers TAAAACACCAGAACTTAGTTTCGACGGATTCTAGAATGCGTAAAGGAGAAGAACTTTTCA and AGGTCGACGGTATCGATAAGCTTGATATCGAATTCTTAAACTGCTGCAGCGTAGTTTTCG. The other eight *eGFP* variants were assembled using IDT’s gBlocks. The *eGFP* genes were cloned into the p41K-GPD plasmid [36] using yeast homologous recombination and overlapping sequences and a high efficiency, lithium-acetate transformation. The formation of correct plasmids was confirmed using DNA sequencing. For each variant, three biological replicates were isolated and stored.

### Growth and media conditions

YPD media contains 20 g/L yeast extract, 10 g/L peptone and 10 g/L glucose. Minimal media contains 6.7 g/L nitrogen base, 20 g/L glucose. Minimal media was supplemented with amino acids; 0.77 g/L of CSM –His (MP Biomedicals) for p413 vectors and 0.79 g/L of CSM for p41K vectors. Media for p41K vectors was supplemented with 200 μg/L of G418. Bacteria were grown in lysogeny broth with ampicillin. All yeast strains were grown at 30°C and bacteria at 37°C. Agar plates were grown in standing incubators and cultures in shakers operating at 225 rpm.

### Flow cytometry

Flow cytometry was used to determine *eGFP* expression. Stationary phase culture was used to inoculate 6 mL of YPD at an OD600 of 0.005. Cells were grown for 12 hours, allowing cultures to reach mid-log phase. Cells were pelleted and re-suspended in cold water. Fluorescent expression profiles were determined using a FACS Fortessa. Forward scattering had a voltage setting of 209 and ampgain of 1.00, side scattering a voltage of 209 and ampgain of 1.00 and fluorescence a voltage of 308 and ampgain of 1.00. Forward and side scattering data were linear and fluorescence was collected on a logarithmic scale. Threshold was set to a forward scattering value of 5000 with an OrOperator and area scaling of 0.71. Gating and statistical analysis of the data was performed using FlowJo 7.6.

### *CatA* activity assay

Yeast minimal media was inoculated at an OD600 of 0.1 using stationary phase cultures of the *CatA* variants. Flasks contained 200 mL, 100 mL and 50 mL of media for the 6, 18 and 24 hour growth experiments respectively. After the designated time period, cells were pelleted and protein was extracted as previously described [22]. Total protein was determined using a Bradford assay. V_max_ values were measured on a microgram of protein basis using a kinetic assay measuring the conversion of added catechol to muconic acid, which can be detected at 288 nm. All biological replicates were included and measurements were done in technical triplicate. Catechol was mixed with protein extract at four concentrations, 0.1, 0.2, 0.3 and 0.4 mM, and Lineweaver-Burke plots were used to calculate V_max_ in units of mM/min*μg protein. A higher V_max_ corresponds to more *CatA* enzyme in the protein extract.

### Muconic acid production

High pressure liquid chromatography (HPLC) was used to measure the intercellular conversion of catechol to muconic acid in *S. cerevisiae* cultures as previously described [22]. Triplicate yeast cultures expressing each *CatA* variant were grown in 30 mL of media for 18 hours with a starting OD600nm of 0.1. After 18 hours, cultures were spiked with 1 mg/mL of catechol and grown for an additional 24 hours. At this point, 1 mL of supernatant was filtered and analyzed using a Zorbax SB-Aq column (Agilent Technologies). The injection volume was 2.0 μL and the mobile phase was 84% 25 mM potassium phosphate buffer (pH = 2.0) and 16% acetonitrile with a flow rate of 1.0 mL/min. The column was maintained at 30°C and the UV–vis absorption was measured at 280 nm. Muconic acid production levels were calculated using a standard curve. *Cis,cis-*muconic acid standards were purchased from Sigma-Aldrich and *cis,trans-*muconic acid was provided by Draths Corporation.

### Matrix drift analysis

The Frobenius matrix norm is defined as the square root of the product of the trace of the conjugate transpose of the matrix and the matrix itself:

$${||A||}_{F}=\sqrt{\overset{m}{\underset{i-1}{\sum }}\overset{n}{\underset{j=1}{\sum }}{\left|a_{\mathrm{ij}}\right|}^{2}}=\;\sqrt{\mathrm{trace}\left(A*A\right)}\;=\sqrt{\sum_{i=1}^{\min\left\{m,n\right\}}\sigma i^{2}}$$

The drift between any two codon usage matrices was determined by taking the difference between the matrices (excluding stop codon usage) and the Frobenius matrix norm of that resultant matrix of differences, or ||A-B||_F_ where A and B represent two distinct matrices of identical size.

The genetic interaction targets for sixteen *S. cerevisiae* transcription factors were identified using the *Saccharomyces* Genome Database (http://yeastgenome.org). Using those corresponding gene target sequences, codon usage matrices were constructed for each transcription factor. Frobenius matrix norms were calculated for all matrix pairs, including the control matrix using MATLAB.

## Competing interests

The authors declare that they have no competing interests.

## Authors’ contributions

AML helped conceive the study, wrote python code, participated in cloning genetic constructs, participated in experiments and drafted the manuscript. KAC participated in experiments and helped draft and edit the manuscript. LGR participated in cloning genetic constructs. HAS helped conceive the study, participated in design and coordination and helped edit the manuscript. All authors read and approved the final manuscript.

## Supplementary Material

Additional file 1 Python script ‘CodonUsageAnalysis’ to read gene sequence(s) as a text file and determines the codon distribution, which is output in a .txt file. Click here for file

Additional file 2 Python script ‘GeneDesign’ Python script to read a protein sequence and a specified codon distribution, using this information to stochastically construct a corresponding gene sequence. Click here for file

Additional file 3 Color-coded 64×64 codon usage matrices for various conditions discussed throughout the manuscript. Click here for file

Additional file 4 Gene sequences, supplementary codon distribution tables. Click here for file
