# Supplementary material for: A condition-specific codon optimization approach for improved heterologous gene expression in Saccharomyces cerevisiae
Source: BMC Syst Biol. 2014 Mar 17;8:33. doi: 10.1186/1752-0509-8-33 (PMC4004289; doi:10.1186/1752-0509-8-33)
Supplement: Additional file 4 — Gene sequences, supplementary codon distribution tables. [file 1752-0509-8-33-S4.pdf]

Supplementary Sequences and Tables for:

A Condition-Specific Codon Optimization Approach for Improved Heterologous Gene Expression in *Saccharomyces cerevisiae*

Amanda M. Lanza[†,§], Kathleen A. Curran[†], Lindsey G. Rey[†] and Hal S. Alper[†,‡,*]

[†] Department of Chemical Engineering

The University of Texas at Austin

200 E Dean Keeton St. Stop C0400

Austin, Texas 78712

[‡] Institute for Cellular and Molecular Biology

The University of Texas at Austin

2500 Speedway Avenue

Austin, Texas 78712

[§]Current Address: Bristol-Myers Squibb

Biologics Development

35 South Street

Hopkinton, Massachusetts 01748

Corresponding Author: * Phone: (512) 471-4417, E-mail: halper@che.utexas.edu
A.M. Lanza: lanza@che.utexas.edu, K.A. Curran: kate.a.curran@gmail.com, L.G. Rey: lindseyr92@aol.com

Gene Variants

All genes were assembled from gBlock fragments ordered from IDT using homologous recombination. Gene sequence was confirmed by standard sequencing.

eGFP WT

ATGCGTAAAGGAGAAGAACTTTTCACTGGAGTTGTCCCAATTCTTGTTGAATTAGAT
GGTGATGTTAATGGGCACAAATTTTCTGTCAGTGGAGAGGGTGAAGGTGATGCAAC
ATACGGAAAACTTACCCTTAAATTTATTTGCACTACTGGAAAACTACCTGTTCCATG
GCCAACACTTGTCACTACTTTCGGTTATGGTGTTCAATGCTTTGCGAGATACCCAGAT
CATATGAAACAGCATGACTTTTTCAAGAGTGCCATGCCCGAAGGTTATGTACAGGAA
AGAACTATATTTTTCAAAGATGACGGGAACTACAAGACACGTGCTGAAGTCAAGTTT
GAAGGTGATACCCTTGTTAATAGAATCGAGTTAAAAGGTATTGATTTTAAAGAAGAT
GGAAACATTCTTGGACACAAATTGGAATACAACTATAACTCACACAATGTATACATC
ATGGCAGACAAACAAAAGAATGGAATCAAAGTTAACTTCAAAATTAGACACAACAT
TGAAGATGGAAGCGTTCAACTAGCAGACCATTATCAACAAAATACTCCAATTGGCG
ATGGCCCTGTCCTTTTACCAGACAACCATTACCTGTCCACACAATCTGCCCTTTCGAA
AGATCCCAACGAAAAGAGAGACCACATGGTCCTTCTTGAGTTTGTAACAGCTGCTGG
GATTACACATGGCATGGATGAACTATACAAAGGCCTGCAGCAAACGACGAAAACT
ACGCTGCAGCAGTTTAA

eGFP control table (CT)

ATGAGAAAAGGTGAAGAATTGTTTACTGGTGTTGTTCCAATTTTGGTTGAATTGGAT
GGTGATGTTAATGGTCATAAATTTTCTGTTTCTGGTGAAGGTGAAGGTGATGCTACT
TATGGTAAATTGACTTTGAAATTTATTTGTACTACTGGTAAATTGCCAGTTCCATGGC
CAACTTTGGTTACTACTTTTGGTTATGGTGTTCAATGTTTTGCTAGATATCCAGATCA
TATGAAACAACATGATTTTTTTAAATCTGCTATGCCAGAAGGTTATGTTCAAGAAAG
AACTATTTTTTTTAAAGATGATGGTAATTATAAAACTAGAGCTGAAGTTAAATTTGA
AGGTGATACTTTGGTTAATAGAATTGAATTGAAAGGTATTGATTTTAAAGAAGATGG
TAATATTTTGGGTCATAAATTGGAATATAATTATAATTCTCATAATGTTTATATTATG
GCTGATAAACAAAAAAATGGTATTAAAGTTAATTTTAAAATTAGACATAATATTGAA
GATGGTTCTGTTCAATTGGCTGATCATTATCAACAAAATACTCCAATTGGTGATGGT
CCAGTTTTGTTGCCAGATAATCATTATTTGTCTACTCAATCTGCTTTGTCTAAAGATC
CAAATGAAAAAAGAGATCATATGGTTTTGTTGGAATTTGTTACTGCTGCTGGTATTA
CTCATGGTATGGATGAATTGTATAAAAGACCAGCTGCTAATGATGAAAATTATGCTG
CTGCTGTTTAA

eGFP high expression table (HT)

ATGAGAAAGGGTGAAGAATTGTTCACAGGTGTGGTGCCAATTTTGGTGGAATTGGA
TGGTGATGTGAATGGTCATAAGTTCTCAGTGTCAGGTGAAGGTGAAGGTGATGCTAC
ATACGGTAAGTTGACATTGAAGTTCATTTGTACAACAGGTAAGTTGCCAGTGCCATG
GCCAACATTGGTGACAACATTCGGTTACGGTGTGCAATGTTTCGCTAGATACCCAGA
TCATATGAAGCAACATGATTTCTTCAAGTCAGCTATGCCAGAAGGTTACGTGCAAGA

AAGAACAATTTTCTTCAAGGATGATGGTAATTACAAGACAAGAGCTGAAGTGAAGT
TCGAAGGTGATACATTGGTGAATAGAATTGAATTGAAGGGTATTGATTTCAAGGAA
GATGGTAATATTTTGGGTCATAAGTTGGAATACAATTACAATTCACATAATGTGTAC
ATTATGGCTGATAAGCAAAAGAATGGTATTAAGGTGAATTTCAAGATTAGACATAA
TATTGAAGATGGTTCAGTGCAATTGGCTGATCATTACCAACAAAATACACCAATTGG
TGATGGTCCAGTGTTGTTGCCAGATAATCATTACTTGTCAACACAATCAGCTTTGTCA
AAGGATCCAAATGAAAAGAGAGATCATATGGTGTTGTTGGAATTCGTGACAGCTGC
TGGTATTACACATGGTATGGATGAATTGTACAAGAGACCAGCTGCTAATGATGAAA
ATTACGCTGCTGCTGTGTAA

eGFP control matrix 1 (C1)

ATGCGAAAAGGCGAGGAACTGTTTACAGGTGTAGTGCCCATTTTGGTGGAGCTAGA
TGGTGACGTTAACGGTCATAAATTCTCGGTATCCGGAGAAGGCGAAGGTGACGCTA
CCTATGGTAAACTCACCTTAAAGTTCATCTGCACAACTGGAAAGCTTCCAGTCCCTT
GGCCCACATTGGTAACGACATTTGGCTATGGTGTACAATGCTTCGCAAGATACCCGG
ATCACATGAAACAACATGACTTTTTCAAGTCTGCTATGCCTGAAGGTTACGTCCAGG
AGCGGACTATTTTCTTTAAGGATGATGGTAATTACAAAACCCGTGCCGAAGTAAAGT
TCGAAGGCGACACGTTGGTAAACCGCATCGAACTTAAAGGCATAGATTTTAAGGAA
GATGGTAATATCTTGGGTCATAAATTAGAATATAACTACAATTCTCATAACGTTTAC
ATTATGGCTGATAAGCAAAAGAACGGAATTAAAGTGAACTTTAAAATCAGACACAA
CATTGAGGATGGTTCTGTTCAATTGGCTGATCATTACCAACAGAATACACCTATCGG
AGACGGCCCAGTTTTACTACCAGATAATCATTACTTAAGTACTCAGTCTGCATTAAG
CAAGGATCCAAATGAGAAGAGAGATCACATGGTTTTGTTGGAATTTGTAACAGCAG
CCGGAATAACACATGGCATGGACGAGTTGTACAAAAGACCTGCGGCAAATGATGAA
AACTATGCAGCTGCTGTATAA

eGFP control matrix 2 (C2)

ATGAGAAAAGGTGAAGAATTGTTCACAGGAGTTGTTCCCATTTTAGTTGAATTAGAC
GGTGATGTAAATGGTCATAAATTTTCTGTTTCCGGCGAAGGAGAGGGAGATGCAAC
GTACGGAAAGTTGACATTGAAGTTTATATGCACCACAGGAAAGCTTCCCGTTCCATG
GCCTACCTTGGTAACGACGTTTGGTTATGGTGTTCAATGCTTTGCTCGATATCCGGAT
CACATGAAGCAGCATGATTTCTTCAAGAGCGCTATGCCCGAAGGGTATGTTCAAGA
AAGAACCATTTTCTTTAAAGATGATGGCAATTATAAGACAAGAGCTGAAGTAAAAT
TCGAGGGAGATACATTGGTTAATCGAATAGAATTAAAGGGTATTGACTTTAAAGAG
GATGGTAATATTCTGGGTCACAAACTTGAATACAATTATAATTCCCATAACGTCTAC
ATAATGGCCGACAAACAAAAGAATGGTATTAAAGTCAATTTCAAAATTCGTCATAA
CATCGAGGACGGCAGCGTCCAATTAGCGGATCACTATCAACAAAATACACCTATAG
GTGACGGTCCCGTGCTTTTACCAGACAACCACTATCTAAGCACTCAATCTGCTCTAT
CGAAAGATCCTAACGAAAAAAGAGATCATATGGTGCTGTTAGAATTTGTCACTGCG
GCTGGTATTACACATGGGATGGACGAACTTTACAAAAGGCCCGCTGCTAATGATGA
AAATTATGCAGCCGCCGTTTAA

eGFP control matrix 3 (C3)

ATGAGAAAGGGCGAAGAGCTATTCACAGGTGTCGTACCAATTTTAGTAGAATTAGA
CGGTGATGTTAACGGGCACAAGTTTTCTGTTTCTGGAGAAGGAGAAGGCGATGCAA

CTTATGGTAAATTGACTTTAAAATTTATTTGCACGACTGGTAAATTGCCAGTTCCATG
GCCAACTTTAGTTACTACATTTGGTTATGGTGTTCAGTGTTTTGCAAGATACCCAGAT
CATATGAAACAACACGATTTCTTTAAATCTGCAATGCCAGAAGGTTACGTTCAAGAA
AGAACTATCTTCTTTAAAGATGATGGCAACTATAAAACAAGAGCAGAGGTTAAGTT
CGAGGGTGATACTTTGGTTAACAGGATAGAACTAAAGGGCATAGATTTCAAGGAAG
ATGGGAATATATTGGGTCACAAGCTAGAATACAATTACAATAGTCATAACGTTTATA
TAATGGCAGACAAGCAAAAAAATGGAATAAAGGTAAATTTTAAAATTAGACACAAT
ATAGAGGATGGCAGCGTTCAATTAGCTGACCATTATCAACAGAATACACCAATTGGT
GATGGCCCAGTTCTACTGCCTGATAATCATTATTTGTCCACTCAAAGTGCCCTGTCTA
AAGATCCAAATGAGAAGCGAGATCATATGGTACTCTTAGAGTTTGTGACTGCAGCTG
GTATCACACATGGGATGGATGAATTATACAAAAGACCAGCTGCAAATGACGAAAAT
TATGCCGCTGCGGTTTAA

eGFP high expression matrix 1 (H1)

ATGAGAAAGGGTGAAGAACTTTTTACAGGTGTTGTTCCAATCTTGGTAGAACTGGAC
GGTGACGTCAACGGTCACAAGTTCTCAGTGTCAGGGGAGGGTGAAGGTGATGCTAC
CTACGGTAAGCTTACTCTAAAGTTCATCTGTACCACGGGGAAATTACCCGTGCCATG
GCCAACTCTAGTTACAACTTTTGGATACGGTGTTCAATGTTTTGCTAGATACCCAGAT
CATATGAAGCAACACGATTTTTTTAAATCAGCGATGCCGGAAGGTTACGTGCAAGA
AAGGACTATCTTTTTCAAAGATGACGGTAACTACAAGACCAGGGCTGAAGTTAAATT
TGAAGGTGACACTCTGGTGAACCGAATAGAATTAAAGGGTATTGATTTCAAGGAAG
ATGGTAACATTTTGGGTCACAAGTTGGAATACAATTATAACTCCCATAACGTTTACA
TTATGGCTGATAAACAAAAGAATGGTATCAAGGTAAATTTTAAAATCAGACACAAC
ATTGAAGACGGTTCCGTACAATTGGCTGATCATTATCAACAAAATACACCTATTGGT
GACGGTCCTGTTTTACTCCCCGATAATCATTATTTGTCCACTCAATCCGCTTTGTCAA
AGGATCCAAATGAAAAGCGTGACCATATGGTGTTACTGGAATTCGTTACTGCTGCAG
GGATCACGCATGGCATGGATGAATTGTATAAGAGACCAGCTGCCAATGACGAAAAC
TACGCTGCCGCCGTTTAA

eGFP high expression matrix 2 (H2)

ATGCGTAAAGGTGAAGAGCTCTTCACAGGTGTCGTTCCAATCTTGGTCGAACTAGAT
GGTGACGTTAACGGTCACAAGTTTTCTGTTTCCGGTGAAGGTGAAGGTGACGCTACC
TATGGGAAGCTTACGCTGAAATTTATCTGTACAACCGGTAAGTTGCCAGTCCCATGG
CCAACTTTGGTAACAACATTTGGCTACGGTGTTCAATGTTTTGCGCGCTACCCAGAT
CATATGAAGCAGCATGATTTCTTTAAAAGCGCCATGCCAGAGGGTTATGTCCAAGAA
AGGACGATATTCTTCAAGGACGACGGTAACTACAAGACCAGGGCTGAAGTTAAATT
TGAAGGCGACACTCTGGTGAATAGAATAGAACTGAAAGGTATCGATTTCAAGGAAG
ATGGTAACATTCTTGGGCATAAACTAGAATACAATTATAACTCCCATAACGTGTATA
TTATGGCTGACAAGCAAAAAAATGGAATCAAGGTTAACTTCAAAATTCGTCATAAC
ATCGAGGACGGTTCTGTCCAATTGGCTGATCATTATCAACAAAACACCCCAATCGGT
GATGGTCCAGTTCTTCTGCCAGATAACCATTACTTGTCAACTCAAAGCGCACTCTCT
AAGGACCCTAATGAAAAGAGAGATCATATGGTTCTTCTCGAGTTCGTCACTGCTGCT
GGTATCACTCACGGTATGGATGAACTATACAAGAGACCAGCTGCTAATGACGAAAA
TTATGCCGCTGCTGTTTAA

eGFP high expression matrix 3 (H3)

ATGCGTAAAGGTGAAGAATTGTTTACAGGTGTTGTCCCAATCTTGGTCGAATTGGAC
GGTGACGTGAATGGGCATAAATTTTCGGTATCTGGGGAGGGTGAAGGTGATGCTAC
CTACGGTAAGTTGACTCTGAAATTTATCTGTACCACAGGCAAGTTGCCGGTACCGTG
GCCCACGCTCGTTACGACGTTTGGCTACGGTGTTCAATGTTTTGCGCGCTACCCAGA
CCACATGAAACAACACGATTTCTTTAAAAGCGCAATGCCGGAAGGCTACGTTCAAG
AAAGGACAATCTTTTTCAAGGACGACGGTAACTACAAAACTAGAGCTGAAGTCAAG
TTTGAAGGTGACACGCTGGTCAACCGTATTGAATTGAAAGGTATTGACTTCAAGGAA
GACGGTAACATTCTTGGACATAAACTCGAATATAACTACAACTCCCACAACGTTTAT
ATCATGGCCGATAAGCAAAAGAATGGTATTAAGGTGAATTTTAAAATTCGTCACAA
CATTGAAGATGGTTCTGTTCAACTAGCTGACCATTACCAACAAAACACTCCAATCGG
TGACGGTCCAGTCTTGCTGCCCGACAACCATTATCTCTCTACACAATCTGCTCTTTCT
AAGGACCCAAATGAAAAAAGGGATCATATGGTATTGTTAGAGTTTGTTACAGCAGC
TGGTATCACGCATGGTATGGACGAACTGTACAAGAGACCAGCGGCAAACGATGAAA
ACTACGCTGCTGCCGTTTAA

CatA wild type

ATGGAAGTTAAAATATTCAATACTCAGGATGTGCAAGATTTTTTACGTGTTGCAAGC
GGACTTGAGCAAGAAGGTGGCAATCCGCGTGTAAAGCAGATCATCCATCGTGTGCT
TTCAGATTTATATAAAGCCATTGAAGATTTGAATATCACTTCAGATGAATACTGGGC
AGGTGTGGCATATTTAAATCAGCTAGGTGCCAATCAAGAAGCTGGTTTACTCTCGCC
AGGCTTGGGTTTTGACCATTACCTCGATATGCGTATGGATGCCGAAGATGCCGCACT
AGGTATTGAAAATGCGACACCACGTACCATTGAAGGCCCGCTATACGTGGCAGGTG
CGCCTGAATCGGTAGGTTATGCGCGCATGGATGACGGAAGTGATCCAAATGGTCAT
ACCCTGATTCTACATGGCACGATCTTTGATGCAGATGGAAAACCTTTACCCAATGCC
AAAGTTGAAATCTGGCATGCCAATACCAAAGGCTTTTATTCACACTTCGACCCAACA
GGCGAGCAGCAGGCGTTCAATATGCGCCGTAGTATTATTACCGATGAAAACGGTCA
GTATCGCGTTCGTACCATTTTGCCTGCGGGTTATGGTTGCCCACCAGAAGGTCCAAC
GCAACAGTTGCTGAATCAGTTGGGCCGTCATGGTAACCGCCCTGCGCACATTCACTA
TTTTGTTTCTGCCGATGGACACCGCAAACTAACTACGCAAATTAATGTGGCTGGCGA
TCCGTACACCTATGACGACTTTGCTTATGCAACCCGTGAAGGCTTGGTGGTTGATGC
AGTGGAACACACCGATCCTGAAGCCATTAAGGCCAATGATGTTGAAGGCCCATTCG
CTGAAATGGTTTTCGATCTAAAATTGACGCGTTTGGTTGATGGTGTAGATAACCAAG
TTGTTGATCGTCCACGTCTAGCGGTGTAA

CatA Blue Heron

ATGGAAGTTAAGATTTTTAACACTCAAGACGTACAAGATTTTTTACGTGTCGCAAGC
GGATTAGAACAAGAAGGCGGAAATCCCAGAGTAAAGCAAATAATACACAGAGTTTT
ATCAGATTTGTACAAAGCGATAGAAGATTTAAATATAACTTCAGATGAATATTGGGC
TGGTGTAGCATACTTAAATCAATTAGGAGCAAATCAAGAAGCAGGATTATTATCACC
CGGACTAGGTTTCGATCATTATTTAGATATGAGAATGGACGCAGAAGACGCAGCCTT
AGGTATTGAAAACGCCACGCCAAGAACAATAGAAGGACCACTTTATGTTGCAGGTG

CCCCCGAATCAGTAGGTTACGCAAGAATGGATGACGGTTCCGACCCAAATGGCCAC
ACTTTAATTTTACACGGAACAATTTTTGACGCTGATGGTAAACCCCTTCCTAATGCTA
AAGTTGAGATATGGCACGCAAACACTAAAGGTTTCTATTCACATTTTGACCCAACAG
GAGAACAACAAGCATTCAACATGAGAAGATCAATTATAACAGACGAGAACGGACA
ATACAGAGTAAGGACTATATTACCAGCAGGATACGGTTGCCCGCCAGAAGGCCCAA
CACAACAATTACTAAATCAATTAGGTAGACATGGAAATAGACCCGCTCACATTCATT
ATTTTGTTAGCGCAGATGGACACAGGAAATTGACCACACAAATCAATGTTGCAGGA
GATCCCTATACTTACGACGATTTTGCATACGCTACAAGAGAAGGGCTAGTAGTAGAC
GCAGTAGAGCATACAGATCCAGAAGCAATAAAAGCAAATGACGTAGAAGGACCATT
CGCAGAAATGGTTTTCGACCTAAAACTTACTAGATTAGTAGATGGAGTAGATAATCA
AGTTGTAGACAGACCAAGATTAGCAGTCTAA

CatA control 1 (C1)

ATGGAAGTTAAGATTTTTAATACCCAAGATGTGCAGGATTTTTTGAGAGTTGCTTCG
GGCCTAGAACAAGAAGGTGGTAATCCTCGTGTTAAACAGATTATACACCGTGTCTTG
TCCGATCTATATAAAGCAATTGAAGATTTGAACATTACGTCAGATGAATATTGGGCT
GGCGTAGCGTATTTGAACCAGTTAGGTGCTAACCAAGAGGCAGGCTTGTTAAGTCCC
GGTTTGGGCTTTGATCATTACTTGGACATGAGGATGGATGCAGAAGATGCTGCATTA
GGTATTGAAAATGCCACGCCAAGAACTATAGAAGGTCCACTTTATGTTGCAGGTGCC
CCAGAAAGCGTCGGTTACGCTCGTATGGATGATGGATCTGACCCAAATGGACACAC
CTTAATCTTGCACGGAACAATCTTTGATGCAGACGGAAAACCTCTTCCGAACGCAAA
AGTGGAAATTTGGCATGCAAACACTAAGGGCTTTTACAGCCACTTTGACCCAACTGG
TGAGCAGCAGGCATTTAACATGCGAAGAAGTATAATAACTGATGAAAACGGACAAT
ACAGAGTGAGGACCATCTTGCCAGCAGGTTACGGATGTCCTCCAGAGGGTCCCACA
CAACAACTACTTAACCAGTTAGGACGCCATGGTAATAGACCTGCTCATATTCATTAC
TTTGTCTCTGCGGACGGCCATAGAAAGTTAACAACACAAATAAACGTTGCGGGTGAT
CCTTACACTTATGACGACTTCGCATATGCCACCCGTGAGGGCTTAGTTGTAGATGCT
GTCGAACACACTGATCCAGAAGCTATTAAGGCTAATGACGTAGAAGGTCCTTTTGCG
GAAATGGTTTTCGATTTAAAATTAACAAGATTAGTCGATGGAGTTGATAACCAAGTT
GTTGATAGGCCACGACTTGCTGTCTAA

CatA control 2 (C2)

ATGGAAGTGAAAATCTTCAACACACAGGACGTACAGGACTTTTTGAGAGTGGCATC
TGGTTTGGAACAAGAAGGGGGCAATCCTCGAGTGAAGCAGATCATTCATAGAGTGC
TTTCTGATCTATACAAAGCTATCGAAGATTTAAATATTACGTCAGACGAATATTGGG
CAGGGGTCGCTTATTTGAATCAATTAGGTGCCAACCAAGAGGCAGGCCTTTTGAGTC
CAGGATTGGGATTTGACCATTACTTGGATATGCGTATGGATGCTGAGGATGCAGCAT
TAGGAATAGAGAATGCAACACCCAGAACGATAGAGGGACCGTTATATGTTGCTGGT
GCTCCAGAGTCAGTTGGTTACGCCCGTATGGATGATGGCTCTGATCCAAATGGCCAT
ACATTAATTTTGCATGGTACTATATTTGATGCTGATGGAAAACCACTTCCCAACGCT
AAAGTTGAAATTTGGCATGCCAACACCAAGGGGTTTTATTCACACTTTGATCCGACA
GGCGAGCAACAAGCTTTTAACATGAGACGGAGTATAATAACAGATGAAAATGGCCA
GTATAGGGTAAGGACTATTCTACCAGCCGGTTACGGATGTCCCCCAGAAGGTCCCAC
ACAACAATTACTAAACCAACTGGGACGACATGGAAATAGGCCAGCTCATATACACT

ACTTCGTTAGCGCTGATGGCCATAGAAAACTAACAACACAAATAAATGTGGCAGGA
GACCCTTACACATATGACGACTTTGCATATGCCACACGTGAAGGCTTAGTTGTTGAC
GCTGTCGAACATACAGATCCAGAAGCTATCAAGGCTAATGACGTCGAGGGTCCTTTT
GCAGAAATGGTATTTGATTTAAAGTTAACTAGACTAGTGGATGGGGTTGACAATCAA
GTAGTAGATCGCCCCAGATTGGCGGTGTAA

CatA control 3 (C3)

ATGGAGGTAAAAATATTCAACACACAAGATGTTCAAGATTTTTTGAGAGTGGCTTCT
GGCTTAGAGCAAGAAGGTGGGAACCCAAGAGTCAAACAAATAATACACCGAGTGCT
GTCAGATTTATACAAAGCTATTGAAGATCTCAATATAACAAGCGATGAATATTGGGC
CGGCGTGGCATACCTAAACCAATTAGGTGCCAATCAAGAGGCTGGTCTTCTGAGCCC
AGGCCTTGGGTTTGATCATTACTTAGACATGAGGATGGATGCTGAGGACGCAGCATT
AGGGATAGAAAATGCGACTCCAAGAACTATAGAGGGCCCACTATATGTAGCCGGCG
CACCCGAAAGTGTGGGATATGCAAGAATGGATGACGGCTCTGATCCGAACGGTCAT
ACTTTGATACTTCACGGCACCATTTTTGATGCCGATGGCAAGCCATTACCGAATGCG
AAGGTTGAAATTTGGCACGCTAACACTAAGGGCTTTTATTCCCATTTTGATCCTACA
GGAGAACAACAAGCTTTTAACATGAGAAGATCAATAATCACCGACGAGAATGGCCA
ATATAGGGTTAGAACAATATTACCAGCTGGCTACGGTTGTCCTCCTGAGGGCCCGAC
CCAACAGCTCCTTAATCAGTTAGGTCGTCATGGTAACAGACCAGCTCATATACACTA
TTTTGTCAGTGCAGATGGACATCGGAAATTAACTACTCAAATAAACGTAGCAGGCG
ATCCGTACACATATGACGATTTCGCCTATGCAACAAGGGAAGGTCTTGTGGTTGACG
CTGTAGAGCATACCGACCCTGAAGCAATTAAAGCGAATGACGTTGAGGGTCCTTTCG
CCGAAATGGTGTTTGATTTAAAGTTAACTAGACTGGTGGATGGCGTTGATAATCAAG
TTGTAGATCGCCCTAGGCTCGCGGTGTAA

CatA stationary 1 (S1)

ATGGAAGTAAAGATCTTCAACACTCAAGACGTTCAAGATTTTTAAGAGTTGCTTCA
GGACTTGAACAGGAAGGCGGTAACCCTCGGGTAAAGCAAATTATCCATCGGGTCCT
GTCTGATCTATACAAGGCAATCGAAGACCTAAACATCACTTCTGACGAATATTGGGC
GGGCGTGGCGTACCTTAACCAATTGGGAGCGAATCAAGAGGCTGGCTTATTAAGCC
CAGGCCTTGGATTCGATCACTATCTTGATATGAGAATGGACGCAGAAGATGCAGCCT
TAGGTATAGAGAACGCTACCCCAAGAACTATCGAAGGCCCATTGTATGTCGCTGGTG
CCCCCGAGAGTGTCGGTTATGCCCGTATGGATGATGGGTCGGATCCAAACGGTCATA
CTTTGATATTGCACGGTACTATATTCGATGCCGATGGTAAACCTCTGCCTAATGCAA
AGGTGGAAATATGGCATGCGAATACAAAGGGATTCTACTCACATTTTGACCCAACG
GGAGAACAACAAGCCTTCAATATGCGGCGGTCTATTATAACGGATGAGAACGGCCA
ATACAGGGTAAGGACCATATTGCCCGCAGGGTACGGCTGCCCACCAGAAGGTCCAA
CTCAACAACTCTTAAACCAATTGGGCAGGCATGGCAACAGGCCTGCCCACATTCACT
ATTTCGTGTCAGCGGATGGTCACAGGAAGTTAACAACACAAATCAACGTCGCAGGT
GATCCGTACACCTACGACGATTTTGCATATGCTACCAGAGAAGGCCTTGTAGTTGAT
GCTGTGGAACATACGGACCCCGAAGCGATCAAGGCCAATGATGTAGAAGGTCCTTT
CGCGGAGATGGTTTTCGATTTGAAATTGACGAGACTAGTTGATGGTGTAGATAATCA
GGTTGTAGACAGACCAAGGTTAGCAGTCTAA

CatA stationary 2 (S2)

ATGGAAGTTAAAATCTTCAACACCCAGGATGTTCAAGACTTTTTGCGTGTAGCCTCC
GGACTTGAACAAGAAGGTGGTAATCCAAGAGTAAAGCAGATCATTCACAGAGTTTT
ATCTGATCTATACAAGGCGATCGAAGATTTGAATATCACTTCGGACGAGTACTGGGC
CGGAGTTGCTTACTTGAATCAGTTGGGTGCTAACCAAGAAGCCGGTTTGTTGTCACC
AGGCTTGGGTTTTGACCATTACCTCGACATGCGGATGGATGCTGAAGACGCGGCCCT
GGGTATTGAAAACGCTACCCCAAGGACGATAGAAGGCCCCCTTTATGTTGCAGGTG
CTCCTGAGAGTGTGGGCTATGCAAGAATGGACGACGGTTCTGACCCCAACGGTCAC
ACATTGATTTTGCACGGTACAATCTTTGACGCCGATGGTAAGCCGTTGCCAAACGCT
AAGGTGGAGATTTGGCATGCGAATACCAAAGGTTTTTATAGCCACTTCGATCCAACA
GGAGAACAACAAGCTTTCAATATGAGAAGATCGATTATTACAGACGAAAACGGACA
ATATAGAGTCAGGACTATACTGCCCGCGGGATACGGTTGTCCTCCAGAAGGTCCAAC
GCAGCAACTACTTAATCAATTAGGAAGGCATGGAAATAGACCCGCTCATATCCACT
ATTTCGTTTCTGCTGATGGCCATCGTAAATTGACTACTCAAATCAACGTTGCCGGTG
ATCCATATACTTATGATGACTTTGCCTATGCAACACGAGAAGGCTTAGTAGTGGACG
CTGTGGAGCACACCGACCCTGAAGCTATCAAAGCTAACGACGTTGAAGGGCCTTTC
GCGGAAATGGTCTTTGACTTGAAACTAACCAGATTAGTCGACGGAGTAGATAACCA
AGTTGTGGACCGGCCTCGATTAGCAGTCTAA

CatA stationary 3 (S3)

ATGGAGGTTAAGATTTTCAACACTCAAGATGTCCAAGACTTTTTAAGAGTGGCCTCG
GGGCTGGAACAAGAAGGCGGCAATCCAAGAGTTAAACAGATCATCCATAGAGTTTT
GTCCGATCTTTACAAAGCCATTGAAGATTTAAACATCACTTCAGACGAATATTGGGC
AGGAGTAGCTTACTTGAATCAGTTGGGTGCTAACCAGGAAGCCGGTCTGCTATCTCC
TGGCCTAGGTTTCGATCACTATTTGGATATGAGAATGGATGCTGAAGATGCAGCATT
AGGTATCGAGAATGCTACTCCAAGAACGATAGAAGGGCCTCTATATGTAGCAGGTG
CTCCCGAGTCGGTCGGCTACGCCCGTATGGACGACGGTTCAGATCCGAACGGACAT
ACTCTGATTCTACATGGAACAATCTTTGACGCCGATGGAAAGCCCCTTCCCAACGCT
AAAGTTGAAATCTGGCATGCCAATACTAAGGGATTTTATTCGCACTTCGATCCCACT
GGTGAACAACAGGCTTTCAATATGAGGCGTAGTATCATCACTGATGAGAATGGCCA
ATACAGAGTTAGAACAATATTACCCGCGGGATACGGGTGTCCTCCTGAAGGACCCA
CTCAACAATTACTCAACCAATTAGGTAGACATGGTAACCGCCCTGCTCATATTCACT
ACTTTGTGTCCGCAGACGGTCACCGTAAGTTAACGACACAAATCAACGTTGCCGGTG
ACCCGTACACTTACGACGATTTCGCCTACGCTACTAGAGAGGGTTTAGTTGTCGATG
CTGTAGAACATACTGATCCGGAAGCTATTAAAGCAAATGACGTTGAAGGGCCATTT
GCAGAGATGGTTTTTGACTTGAAACTGACACGGTTAGTCGATGGGGTGGACAACCA
AGTGGTTGATAGACCCAGGCTCGCAGTCTAA

CatA high expression 1 (H1)

ATGGAAGTCAAGATTTTTAATACTCAAGATGTCCAAGATTTCTTGAGAGTTGCCTCT
GGACTGGAACAAGAGGGTGGTAACCCAAGAGTCAAGCAAATCATCCACCGTGTCTT
GTCTGATTTGTACAAGGCCATCGAAGATTTAAATATTACGTCGGACGAATACTGGGC
AGGCGTTGCCTACTTGAACCAGTTAGGTGCTAACCAAGAAGCCGGTCTATTATCACC

AGGTTTGGGTTTCGACCACTACTTGGACATGAGAATGGATGCTGAAGACGCTGCTCT
GGGAATCGAAAACGCTACCCCAAGAACCATTGAAGGTCCACTGTACGTCGCCGGCG
CTCCAGAGTCTGTGGGTTACGCTAGAATGGATGATGGCTCTGACCCAAATGGTCATA
CGTTGATTCTTCATGGCACGATATTTGACGCTGACGGTAAGCCACTGCCAAATGCCA
AGGTTGAAATCTGGCATGCTAATACTAAGGGTTTCTATTCACATTTCGATCCAACCG
GTGAACAACAAGCTTTCAATATGAGAAGGTCGATTATAACTGATGAAAACGGTCAA
TACAGAGTTAGAACAATCCTACCAGCTGGTTACGGTTGCCCACCAGAGGGTCCAACT
CAACAATTACTGAATCAGTTAGGTAGACACGGTAACAGACCTGCGCATATCCATTAT
TTTGTGTCGGCTGATGGGCACAGAAAGCTAACAACTCAAATCAACGTGGCCGGTGA
CCCATACACCTACGACGACTTCGCCTACGCCACTCGGGAAGGTCTGGTGGTAGATGC
TGTTGAACACACCGACCCAGAGGCCATTAAAGCTAACGATGTGGAGGGTCCATTCG
CCGAAATGGTTTTCGATCTGAAACTGACCAGGCTTGTAGATGGTGTTGACAACCAAG
TTGTGGACAGACCAAGACTTGCGGTTTAA

CatA high expression 2 (H2)

ATGGAAGTCAAAATTTTCAACACGCAGGATGTCCAAGATTTCTTGAGGGTAGCTTCT
GGGTTAGAACAAGAGGGTGGTAACCCAAGAGTCAAGCAAATCATCCACAGAGTTTT
GTCAGATCTGTACAAAGCTATCGAAGATTTAAATATCACAAGTGATGAATACTGGGC
TGGTGTCGCTTACCTTAATCAGCTAGGCGCTAACCAAGAAGCTGGTTTATTGTCTCC
GGGGTTAGGTTTCGACCACTACTTAGACATGCGTATGGACGCTGAAGATGCCGCTTT
GGGTATCGAAAATGCCACCCCAAGAACTATCGAAGGTCCACTGTATGTGGCGGGGG
CACCAGAATCAGTGGGATACGCTAGAATGGATGACGGTTCTGACCCAAATGGGCAT
ACGTTGATTCTACATGGTACCATCTTCGATGCTGATGGTAAGCCTCTTCCTAACGCTA
AAGTAGAAATCTGGCATGCTAACACAAAGGGTTTTTACTCTCATTTCGATCCAACGG
GGGAGCAACAAGCTTTCAATATGCGTCGCAGCATCATCACCGACGAAAATGGACAA
TACAGGGTCAGAACAATATTGCCTGCGGGTTACGGTTGCCCCCCAGAAGGTCCAAC
GCAGCAATTGTTGAACCAATTGGGTAGACATGGCAATAGACCAGCTCACATTCACTA
CTTTGTCTCCGCTGATGGTCACAGAAAGTTGACCACACAAATCAACGTTGCCGGTGA
CCCATACACTTACGATGACTTTGCATACGCTACCAGAGAAGGTTTGGTTGTTGACGC
TGTTGAACACACGGACCCAGAAGCTATCAAGGCCAATGACGTCGAAGGTCCATTCG
CCGAAATGGTTTTCGACTTGAAGCTAACCAGACTGGTAGATGGTGTAGATAATCAGG
TGGTCGATCGTCCCCGATTAGCTGTCTAA

Table S1

Control codon usage table. A control codon usage table was generated from the sequences of 6,666 protein-coding genes in *S. cerevisiae* using the CodonUsageBias python script. From these sequences, we are able to determine for all 64 codons the total count of each codon, frequency of occurrence per 1000 codons, and probability amongst synonymous codons (fraction).

Amino	Codon	Count	Frequency per 1000	Fraction
Ala	GCG	18708	6.22	0.11
	GCA	48873	16.25	0.30
	GCT	60549	20.13	0.37
	GCC	36389	12.1	0.22
Cys	TGT	24477	8.14	0.62
	TGC	15123	5.03	0.38
Asp	GAT	112614	37.44	0.65
	GAC	60473	20.1	0.35
Glu	GAG	58013	19.29	0.30
	GAA	135489	45.04	0.70
Phe	TTT	80562	26.78	0.59
	TTC	54877	18.24	0.41
Gly	GGG	18301	6.08	0.12
	GGA	33737	11.22	0.23
	GGT	67010	22.28	0.45
	GGC	29538	9.82	0.20
His	CAT	41902	13.93	0.64
	CAC	23507	7.81	0.36
Ile	ATA	55646	18.5	0.28
	ATT	90225	29.99	0.46
	ATC	51005	16.96	0.26
Lys	AAG	90474	30.08	0.41
	AAA	127843	42.5	0.59
Leu	TTG	79567	26.45	0.28
	TTA	79072	26.29	0.28
	CTG	32360	10.76	0.11
	CTA	40619	13.5	0.14
	CTT	38311	12.74	0.13
	CTC	17438	5.8	0.06
Met	ATG	62753	20.86	1
Asn	AAT	109078	36.26	0.60
	AAC	73921	24.57	0.40
Pro	CCG	16471	5.48	0.12
	CCA	53446	17.77	0.41
	CCT	40872	13.59	0.31
	CCC	20970	6.97	0.16
Gln	CAG	37106	12.34	0.32

Amino	Codon	Count	Frequency per 1000	Fraction
	CAA	80576	26.79	0.68
Arg	AGG	28378	9.43	0.21
	AGA	62777	20.87	0.47
	CGG	5765	1.92	0.04
	CGA	9673	3.22	0.07
	CGT	19062	6.34	0.14
	CGC	8225	2.73	0.06
Ser	AGT	43985	14.62	0.16
	AGC	30320	10.08	0.11
	TCG	26613	8.85	0.10
	TCA	57738	19.19	0.21
	TCT	70764	23.53	0.26
	TCC	42720	14.2	0.16
Thr	ACG	24689	8.21	0.14
	ACA	54561	18.14	0.31
	ACT	60493	20.11	0.34
	ACC	37621	12.51	0.21
Val	GTG	32469	10.79	0.19
	GTA	36737	12.21	0.22
	GTT	64463	21.43	0.38
	GTC	33842	11.25	0.20
Trp	TGG	31313	10.41	1
Tyr	TAT	57528	19.12	0.57
	TAC	43710	14.53	0.43

Table S2
High expression codon usage table. A high expression codon usage table was generated from the sequences 100 most highly expressed protein-coding genes in *S. cerevisiae* using the CodonUsageBias python script. From these sequences, we are able to determine for all 64 codons the total count of each codon, frequency of occurrence per 1000 codons, and probability amongst synonymous codons (fraction).

Amino Acid	Codon	Count	Frequency per 1000	Fraction
Ala	GCG	19	1.02	0.04
	GCA	22	1.18	0.05
	GCT	299	16.01	0.65
	GCC	118	6.32	0.26
Cys	TGT	256	13.71	0.68
	TGC	119	6.37	0.32
Asp	GAT	258	13.81	0.53
	GAC	227	12.15	0.47
Glu	GAG	215	11.51	0.28
	GAA	553	29.61	0.72
Phe	TTT	212	11.35	0.46
	TTC	245	13.12	0.54
Gly	GGG	58	3.11	0.12
	GGA	47	2.52	0.10
	GGT	345	18.47	0.73
	GGC	25	1.34	0.05
His	CAT	140	7.5	0.55
	CAC	113	6.05	0.45
Ile	ATA	62	3.32	0.14
	ATT	195	10.44	0.44
	ATC	188	10.07	0.42
Lys	AAG	944	50.54	0.61
	AAA	592	31.7	0.39
Leu	TTG	788	42.19	0.26
	TTA	308	16.49	0.10
	CTG	773	41.39	0.25
	CTA	442	23.67	0.14
	CTT	523	28	0.17
	CTC	234	12.53	0.08
Met	ATG	332	17.78	1
Asn	AAT	341	18.26	0.50
	AAC	335	17.94	0.50
Pro	CCG	296	15.85	0.26
	CCA	610	32.66	0.53
	CCT	168	9	0.14
	CCC	86	4.6	0.07
Gln	CAG	198	10.6	0.36

Amino Acid	Codon	Count	Frequency per 1000	Fraction
	CAA	348	18.63	0.64
Arg	AGG	427	22.86	0.37
	AGA	663	35.5	0.58
	CGG	10	0.54	0.01
	CGA	6	0.32	0.01
	CGT	34	1.82	0.03
	CGC	3	0.16	<0.01
Ser	AGT	208	11.14	0.11
	AGC	119	6.37	0.06
	TCG	360	19.28	0.19
	TCA	490	26.24	0.26
	TCT	472	25.27	0.25
	TCC	259	13.87	0.14
Thr	ACG	449	24.04	0.27
	ACA	504	26.99	0.30
	ACT	421	22.54	0.25
	ACC	300	16.06	0.18
Val	GTG	510	27.31	0.30
	GTA	390	20.88	0.23
	GTT	467	25	0.27
	GTC	340	18.2	0.20
Trp	TGG	433	23.18	1
Tyr	TAT	80	4.28	0.32
	TAC	169	9.05	0.68
